# Paralysis to Analysis: Unmasking Thyrotoxic Periodic Paralysis in a Middle-Aged Male Patient With Undiagnosed Graves Disease

**DOI:** 10.7759/cureus.104938

**Published:** 2026-03-09

**Authors:** Joseph A White, Sofia Dinges, Elham Shams, Sarah Nickle, Patrick Landayan

**Affiliations:** 1 Internal Medicine, Florida International University, Herbert Wertheim College of Medicine, Miami, USA; 2 Internal Medicine, Miami Veterans Affairs Healthcare System, Miami, USA; 3 Internal Medicine, Nova Southeastern University Dr. Kiran C. Patel College of Osteopathic Medicine, Fort Lauderdale, USA; 4 Translational Medicine, Florida International University, Herbert Wertheim College of Medicine, Miami, USA; 5 Pharmacology and Therapeutics, Miami Veterans Affairs Healthcare System, Miami, USA

**Keywords:** endocrinology, flaccid paralysis, graves disease, hyperthyroidism, hypokalemia, muscle weakness, periodic paralysis, thyrotoxicity, thyrotoxic period paralysis, weakness

## Abstract

Thyrotoxic periodic paralysis (TPP) is a rare complication of hyperthyroidism characterized by sudden-onset muscle weakness and hypokalemia due to intracellular potassium shifts. It most commonly affects young Asian men and is rarely seen in older adults or non-Asian populations. We present the case of a 52-year-old Hispanic male patient who experienced episodic bilateral limb weakness and falls over several months, ultimately diagnosed as TPP secondary to previously undiagnosed Graves' disease. Despite normal neurologic imaging and no overt hyperthyroid symptoms, thyroid function testing revealed severe thyrotoxicosis. Treatment with potassium replacement, methimazole, and propranolol led to the resolution of symptoms. This case highlights the importance of maintaining a high index of suspicion for TPP in patients with recurrent weakness, even when demographic and clinical features are atypical.

## Introduction

Thyrotoxic periodic paralysis (TPP) is a rare cause of acute flaccid paralysis resulting from underlying thyrotoxicosis. It is characterized by episodic, painless muscle weakness associated with hypokalemia due to intracellular potassium shifts driven by excess thyroid hormone [1]. Attacks classically involve the proximal muscles, occur abruptly, and often develop at night or in the early morning hours, frequently precipitated by high-carbohydrate intake, alcohol consumption, or rest following strenuous exercise.

TPP is a subtype of hypokalemic periodic paralysis and represents one of the two dominant forms of periodic paralysis in Asian populations, whereas familial hypokalemic periodic paralysis predominates in Western countries [2]. Although TPP most commonly affects young Asian men between 20 and 40 years of age, it is rarely reported in older adults or non-Asian populations, contributing to frequent diagnostic delay [3]. Notably, overt signs of hyperthyroidism are present in only 17-50% of cases at initial presentation, further obscuring recognition [4].

Clinically, TPP may mimic other causes of acute flaccid paralysis such as Guillain-Barré syndrome, spinal cord compression, primary hypokalemic periodic paralysis, or myasthenia gravis, making early diagnostic consideration critical [4,5]. Delayed or missed diagnosis of TPP carries significant clinical consequences, including life-threatening cardiac arrhythmias, respiratory muscle involvement, and unnecessary neurologic imaging or invasive testing. In this report, we describe the case of a 52-year-old Hispanic male patient with recurrent episodes of weakness and falls initially attributed to chronic lumbar spine disease, ultimately diagnosed with TPP secondary to previously unrecognized Graves' disease.

## Case presentation

A 52-year-old Hispanic male Veteran with allergic rhinitis, migraines, and insomnia reported to the Emergency Department (ED) with significant back pain and difficulty ambulating. He reported that due to significant weakness in his legs the previous night, he experienced a fall in which he was unable to get up on his own. Significantly, at presentation, the patient was unable to straighten either leg. 

He denied any loss of consciousness, syncope, loss of sensation in the perianal region, urinary/fecal incontinence, or retention associated with the fall. On admission, the patient reported that he had dealt with low back pain since 2007 after sustaining an injury in Iraq. Upon chart review, the patient underwent a lumbar discectomy in 2019, in which the patient reports improved symptoms for several years, until recently. Over the several months preceding presentation, the patient reported intermittent episodes of sudden-onset bilateral lower extremity weakness, occasionally involving the upper extremities, lasting several hours and resolving spontaneously. Episodes occurred unpredictably, including overnight and early morning hours, and were not consistently associated with exertion, high-carbohydrate meals, alcohol intake, or recent illness. Between episodes, he returned to near-baseline strength but noted increasing frequency of attacks and progressive difficulty ambulating, culminating in the fall that prompted hospital presentation. Relevant home medications at the time of presentation included cyclobenzaprine 10 mg orally three times daily, diclofenac 1% topical gel three times daily as needed, gabapentin 100 mg orally three times daily, and ibuprofen 800 mg orally three times daily as needed. The patient denied a family history of thyroid disease.

Examinations

On presentation to the ED, the patient was afebrile with a temperature of 36.5 °C, tachycardic with a heart rate of 113 beats per minute, normotensive with a blood pressure of 131/77 mmHg, a respiratory rate of 16 breaths per minute, and an oxygen saturation of 97% on room air.

Physical examination revealed an alert and oriented male in mild distress due to weakness. Cardiovascular examination demonstrated a regular rhythm without murmurs. Pulmonary examination was unremarkable with clear breath sounds bilaterally. Musculoskeletal examination was notable for an inability to lift the bilateral lower extremities against gravity. There was no paraspinal tenderness, midline spinal tenderness, step-off deformity, or saddle anesthesia.

On inpatient neurologic examination, there were no focal sensory deficits. Motor strength was reduced to 3/5 in both the upper and lower extremities bilaterally. Tongue fasciculations and a fine bilateral hand tremor were observed. Reflexes were preserved, and cranial nerve examination was otherwise normal.

Preliminary Differentials

Given the patient’s acute bilateral weakness and history of lumbar spine disease, the initial differential diagnosis included cauda equina syndrome, cervical or lumbar spinal cord compression, electrolyte abnormalities, myasthenia gravis, and thyrotoxic periodic paralysis.

Investigations

Laboratory Studies

Laboratory evaluation revealed a potassium level of 2.8 mEq/L (Table 1), which increased to 5.1 mEq/L after treatment. Thyroid function tests showed a suppressed thyroid-stimulating hormone (TSH) (<0.01 mIU/L) with an elevated free T4 of 2.9 ng/dL. Additionally, thyroid-stimulating immunoglobulin was positive, consistent with autoimmune hyperthyroidism.

**Table 1:** Laboratory Findings

Laboratory Test	Result	Reference Range	Interpretation
Potassium	2.8 mEq/L	3.5–5.0 mEq/L	Hypokalemia
Potassium (post-treatment)	5.1 mEq/L	3.5–5.0 mEq/L	Normalized
Thyroid-stimulating hormone (TSH)	<0.01 mIU/L	0.4–4.0 mIU/L	Suppressed
Free thyroxine (Free T4)	2.9 ng/dL	0.8–1.8 ng/dL	Elevated
Thyroid-stimulating immunoglobulin	Positive	Negative	Consistent with Graves disease

Imaging

**Figure 1:**
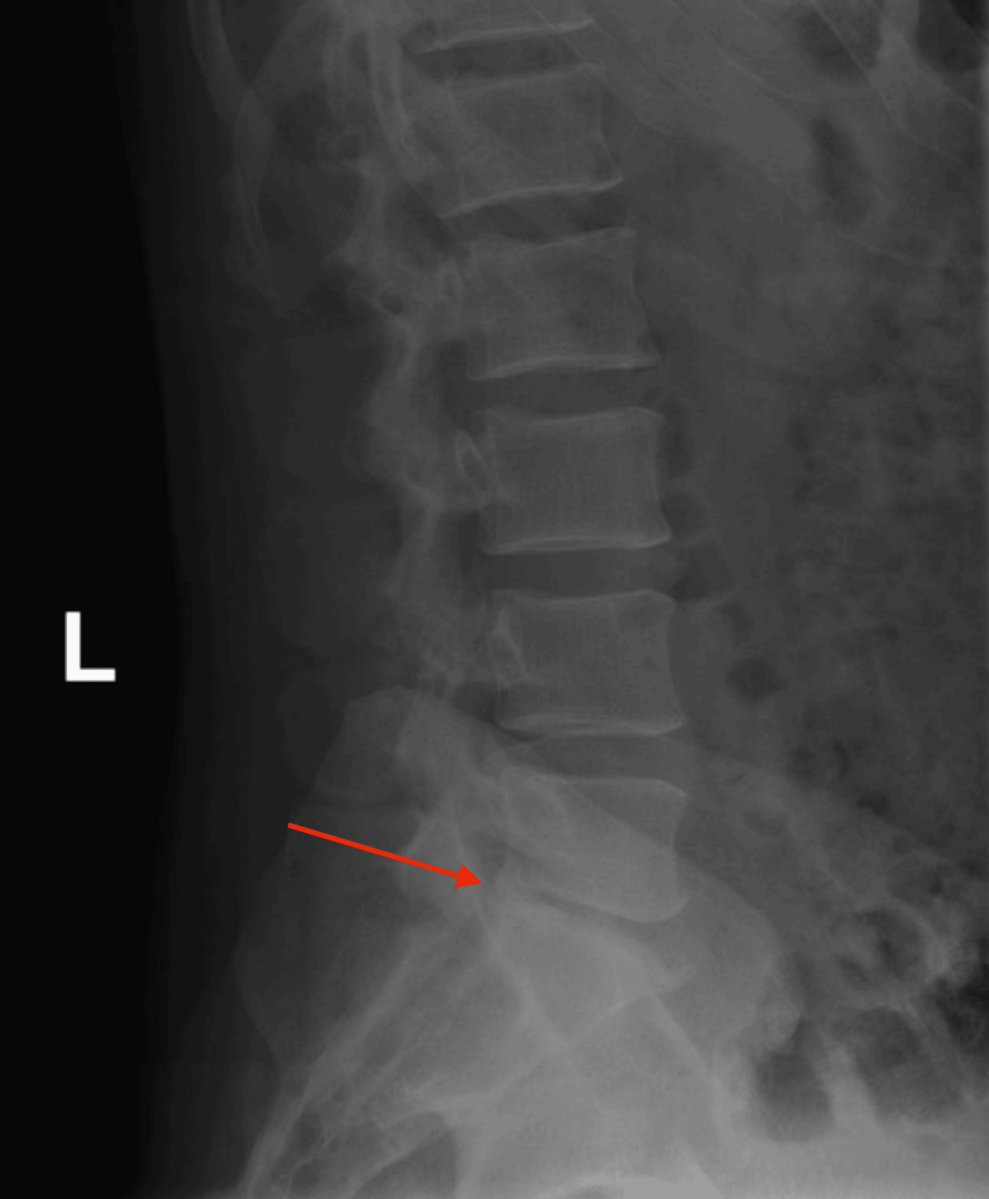
Lateral lumbar spine radiograph demonstrating severe degenerative changes at the L5–S1 disc space (red arrow), including disc space narrowing and endplate sclerosis.

**Figure 2:**
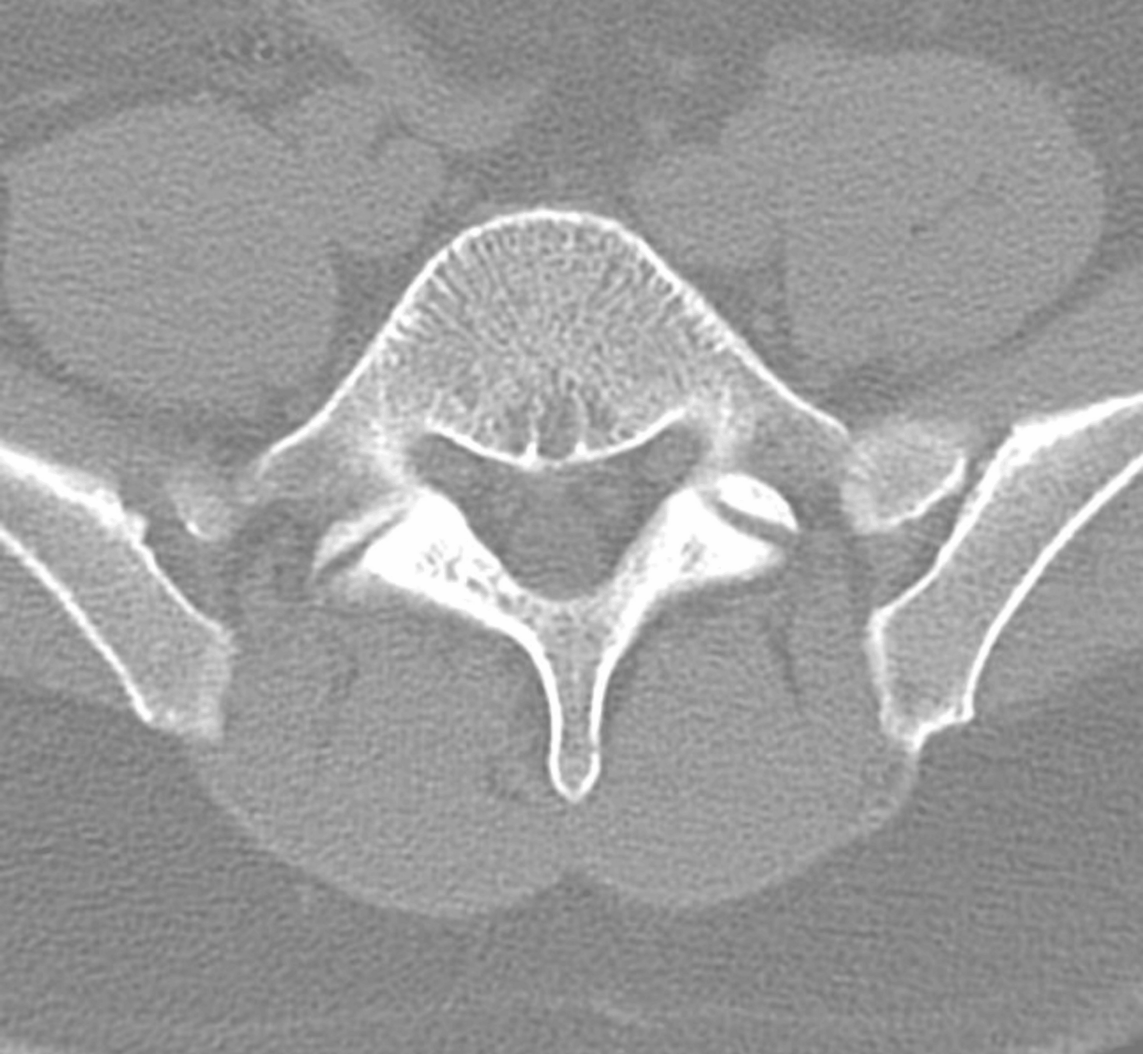
Axial computed tomography image at the L5–S1 level demonstrating degenerative facet arthropathy without evidence of acute fracture, significant spinal canal stenosis, or acute osseous abnormality.

**Figure 3:**
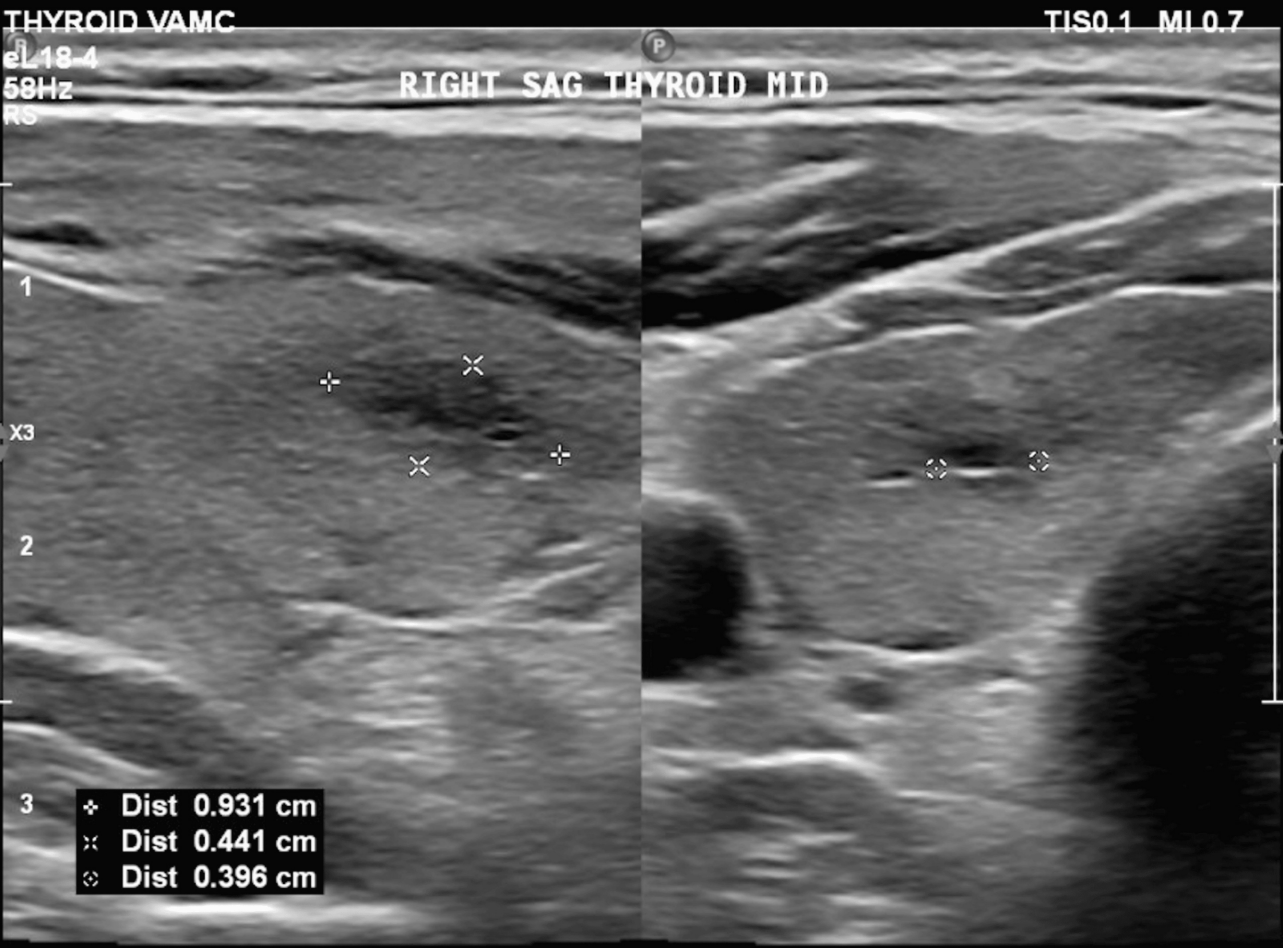
Sagittal mid-right thyroid ultrasound image demonstrating a subcentimeter, ill-defined, mildly hypoechoic area measuring 0.3 × 0.4 × 0.9 cm, without calcifications or taller-than-wide morphology, favored to represent a pseudonodule in the setting of heterogeneous thyroid parenchyma.

**Figure 4:**
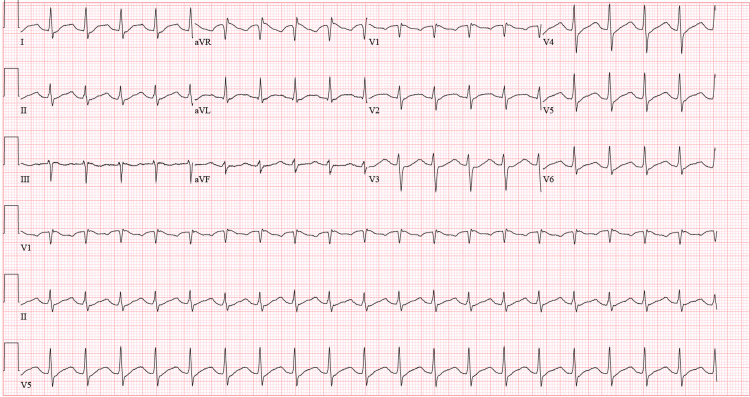
Twelve-lead electrocardiogram demonstrating tachycardia at approximately 120 beats per minute, with narrow QRS duration (98 ms) and prolonged corrected QT interval (QT/QTcB: 430/607 ms). Automated interpretation suggested probable sinus tachycardia with junctional ST-segment depression and QT prolongation.

Initial imaging evaluation included a lateral lumbar spine radiograph, which demonstrated severe degenerative changes at the L5-S1 disc space, characterized by disc space narrowing and endplate sclerosis (Figure 1). An axial computed tomography (CT) image of the lumbar spine at the L5-S1 level revealed degenerative facet arthropathy without evidence of acute fracture, significant spinal canal stenosis, or other acute osseous abnormality (Figure 2). CT of the head, cervical spine, and thoracic spine showed no acute intracranial or spinal pathology. Thyroid ultrasound demonstrated a subcentimeter, ill-defined, mildly hypoechoic area within the mid-right thyroid lobe measuring 0.3 × 0.4 × 0.9 cm, without calcifications or taller-than-wide morphology, favored to represent a pseudonodule in the setting of heterogeneous thyroid parenchyma (Figure 3). A 12-lead electrocardiogram revealed tachycardia at approximately 120 beats per minute, with a narrow QRS duration and prolonged corrected QT interval, with automated interpretation suggesting probable sinus tachycardia with junctional ST-segment depression and QT prolongation (Figure 4).

Primary diagnosis

The primary diagnosis was TPP secondary to newly diagnosed Graves' disease, supported by laboratory findings of suppressed TSH, elevated free T4, positive thyroid-stimulating immunoglobulin, and the clinical presentation of hypokalemia-associated episodic muscle weakness.

Treatment

In the ED, medications to target the patient’s pain and correct hypokalemia were ordered. Regarding pain, the patient received one-time doses of dexamethasone 10 mg IV, ketorolac 30 mg IV, and morphine 4 mg IV. Despite this multimodal pain control approach, the patient was still unable to straighten his legs bilaterally, and bilateral arm and leg weakness persisted. For the treatment of hypokalemia, the patient was given potassium chloride 40 meq orally one time and potassium chloride 10 meq IV one time. The patient was then admitted for further management of intractable, acute-on-chronic low back pain and further evaluation of weakness. 

During admission, as repeat potassium was within normal limits, no further treatment for hypokalemia was indicated. For continued treatment of pain, the patient was ordered acetaminophen 1000 mg IV every eight hours as needed for mild pain, ketorolac 30 mg IV every six hours as needed for moderate pain, and oxycodone 5 mg orally every six hours as needed for severe pain. The patient's home cyclobenzaprine 10 mg orally daily as needed for back spasms was continued during admission. The patient's home gabapentin 100 mg orally three times daily was increased to 300 mg orally three times daily. Once the diagnosis of TPP was established, disease-specific medications were initiated in concert with Endocrinology. Pharmacotherapy for hyperthyroidism followed the 2016 American Thyroid Association Guidelines for Diagnosis and Management of hyperthyroidism and other causes of thyrotoxicosis [6]. The patient was initiated on propranolol 20 mg orally every eight hours. Methimazole 30 mg orally daily was started with plans for repeat labs one week post-discharge and prompt follow-up in the Endocrinology Clinic.

At the time of discharge, the patient was prescribed methimazole 30 mg orally daily and propranolol 20 mg orally every eight hours. Patient was given medications at the bedside at the time of discharge and was counseled regarding appropriate doses and potential adverse effects. The patient was counseled to continue his previous home medications.

Outcome

Upon treatment, bilateral upper and lower extremity strength returned to baseline, tongue fasciculations and hand tremors improved, and tachycardia resolved. The hospital length of stay was three days.

Follow-up

The patient followed up with endocrinology at four months, at which time, he had self-discontinued propranolol. Laboratory testing at follow-up demonstrated a TSH level of 0.15 mIU/L, free T4 of 1.3 ng/dL, and T3 of 0.79 ng/mL. Methimazole increased from 2.5 mg to 5 mg daily due to TSH suppression and continued symptomatology of anxiety and fatigue.

## Discussion

TPP is an uncommon but potentially life-threatening manifestation of thyrotoxicosis characterized by episodic muscle weakness associated with hypokalemia due to intracellular potassium shifts. While TPP is well described in young Asian men, its occurrence in older patients and non-Asian populations remains rare and frequently leads to diagnostic delay [1-3]. This case highlighted an atypical presentation of TPP in a 52-year-old Hispanic male patient whose symptoms were initially confounded by a history of lumbar spine disease.

The pathophysiology of TPP is driven by excess thyroid hormone-mediated upregulation of Na⁺/K⁺-ATPase activity in skeletal muscle, resulting in rapid intracellular potassium shift and subsequent hypokalemia without total body potassium depletion [5]. This disruption of membrane excitability leads to flaccid paralysis, which may occur even at potassium levels that would not typically produce paralysis in primary hypokalemic periodic paralysis [7]. Genetic susceptibility has been implicated, particularly variants involving potassium channels such as KCNJ18, and skeletal muscle calcium-handling proteins including RYR1, suggesting a complex interplay between thyroid hormone excess and ion channel dysfunction [7-9]. 

In this patient, serum potassium increased from 2.8 mEq/L to 5.1 mEq/L following modest potassium repletion, a pattern consistent with rebound hyperkalemia due to reversal of the intracellular potassium shift characteristic of TPP rather than true potassium depletion. This underscores the importance of cautious potassium replacement in suspected TPP, as excessive supplementation may result in transient hyperkalemia once Na⁺/K⁺-ATPase activity normalizes. The patient received a single dose of intravenous dexamethasone prior to diagnostic clarification. While glucocorticoids can influence potassium redistribution and thyroid hormone metabolism, the improvement in neuromuscular symptoms correlated most closely with potassium normalization and initiation of nonselective beta-blockade, suggesting a limited clinical impact from corticosteroid administration in this case.

Importantly, clinical features of hyperthyroidism may be subtle or absent in TPP. Prior studies estimate that overt thyrotoxic symptoms are present in only 17-50% of cases at initial presentation [4]. Consistent with this, our patient lacked classic hyperthyroid complaints, and the diagnosis was only revealed after evaluation for hypokalemia and unexplained weakness. Similar diagnostic challenges have been reported in older and non-Asian patients, where TPP is often misattributed to neurologic or musculoskeletal etiologies [1,3,4].

Our case adds to the growing body of literature demonstrating that TPP should be considered in patients outside the traditional demographic profile, particularly when episodic weakness and hypokalemia are present [1,2,3,10]. Similar presentations with delayed diagnosis have been reported in older and non-Asian patients, where comorbid musculoskeletal or neurologic disease obscured the endocrine etiology, reinforcing the diagnostic challenge highlighted by this case [1,3,4]. Early recognition is critical, as treatment with nonselective beta-blockers and antithyroid therapy leads to rapid symptom resolution and prevents recurrence [5,11]. Potassium replacement should be administered cautiously, as rebound hyperkalemia may occur once the intracellular shift reverses.

This case underscores the importance of maintaining a high index of suspicion for thyrotoxic periodic paralysis in patients presenting with recurrent weakness, even in the presence of alternative explanatory diagnoses such as chronic spine disease. Prompt thyroid function testing in the appropriate clinical context can prevent unnecessary diagnostic procedures and facilitate timely, targeted therapy.

## Conclusions

This case emphasizes the importance of considering TPP in the differential diagnosis of episodic muscle weakness, particularly in patients presenting with hypokalemia, even when they fall outside typical demographic profiles. Although rare in non-Asian and older populations, TPP can present subtly and may be misattributed to musculoskeletal or neurologic conditions. Prompt recognition and treatment with beta-blockers and antithyroid therapy can lead to rapid recovery and prevent recurrence. Clinicians should maintain a high index of suspicion to ensure timely diagnosis and comprehensive management.
